# Roles of E-cadherin and Noncoding RNAs in the Epithelial–mesenchymal Transition and Progression in Gastric Cancer

**DOI:** 10.3390/ijms20122870

**Published:** 2019-06-12

**Authors:** Irina V. Bure, Marina V. Nemtsova, Dmitry V. Zaletaev

**Affiliations:** 1 I.M. Sechenov First Moscow State Medical University (Sechenov University), Moscow 119991, Russia; nemtsova_m_v@mail.ru (M.V.N.); zalnem@mail.ru (D.V.Z.); 2 Research Centre for Medical Genetics, Moskvorechie st., 1, Moscow 115522, Russia

**Keywords:** epithelial–mesenchymal transition, gastric cancer, E-cadherin, microRNAs, long noncoding RNAs

## Abstract

The epithelial–mesenchymal transition (EMT) is thought to be at the root of invasive and metastatic cancer cell spreading. E-cadherin is an important player in this process, which forms the structures that establish and maintain cell–cell interactions. A partial or complete loss of E-cadherin expression in the EMT is presumably mediated by mechanisms that block the expression of E-cadherin regulators and involve the E-cadherin-associated transcription factors. The protein is involved in several oncogenic signaling pathways, such as the Wnt/β-catenin, Rho GTPase, and EGF/EGFR, whereby it plays a role in many tumors, including gastric cancer. Such noncoding transcripts as microRNAs and long noncoding RNAs—critical components of epigenetic control of gene expression in carcinogenesis—contribute to regulation of the E-cadherin function by acting directly or through numerous factors controlling transcription of its gene, and thus affecting not only cancer cell proliferation and metastasis, but also the EMT. This review focuses on the role of E-cadherin and the non-coding RNAs-mediated mechanisms of its expressional control in the EMT during stomach carcinogenesis.

## 1. Introduction

Gastric cancer (GC) is the fifth most common cancer worldwide and the third most deadly. More than a million people were newly diagnosed with GC in 2018 and there were about 783,000 deaths [1]. The incidence and prevalence of GC vary geographically and are the highest in East and Central Asia, Eastern Europe, and Latin America, where 87% of new cases occur. Far lower rates are observed in Africa and North America [2,3].

GC is a multifactorial disease. Both hereditary and environmental factors play an important role in its pathogenesis, including the genetic background of the host, infectious agents, and food habits. Chronic atrophic gastritis and *Helicobacter pylori* infection are the most important risk factors in GC.

According to the Lauren classification, stomach cancer is classified into two types, namely diffuse type and intestinal type. The two types display clinical, morphological, and epidemiological distinctions. The intestinal type is more often found in elderly patients with multifocal atrophic gastritis, which develops into intestinal metaplasia or dysplasia. The diffuse type is more prevalent in younger patients, is more aggressive, and lacks an apparent association with gastritis and metaplasia. Clinical distinctions of the two types are determined by differences in molecular mechanisms of tumor development and progression [4].

Another classification recognizes three types of GC [5]. One includes Lauren’s diffuse GC and signet ring cell carcinoma. The latter is defined as E-cadherin (*CDH1*)-dependent cancer, which is caused by genetic and epigenetic aberrations in E-cadherin. Another type includes distal intestinal tumors, which are associated with atrophic gastritis and *H. pylori* infection. A third type combines proximal tumors of a cardia or gastroesophageal origin, which are associated with life habits and are induced by obesity and chronic reflux of gastric acid. The incidence of the third type is currently increasing worldwide [6].

Four molecular GC subtypes have recently been identified in genomic studies employing whole-genome platforms in protein, transcript, microRNA, CpG methylation, and mutation profiling. The subtypes are microsatellite unstable, Epstein–Barr virus positive, chromosome unstable, and genomically stable. Their validation with clinical patient samples is now in progress [7].

The molecular pathogenesis of GC is to a great extent associated with E-cadherin, which harbor both genetic and epigenetic abnormalities in both germline and sporadic GCs. An extremely broad range of molecular alterations has been observed for *CDH1*, which codes for E-cadherin. The range includes changes in gene expression levels, germline and somatic mutations, allelic deletions of locus 16q22.1, and epigenetic gene silencing via methylation of the promoter region or regulation by noncoding RNAs (ncRNAs).

During the last decades, the ncRNAs have been recognized as an important functional part of the genome. Despite lacking protein-coding capacity, they represent one of the biggest classes of transcriptional and posttranscriptional regulators of gene expression [8,9]. They are expressed in all eukaryotic cells at different stages of ontogenesis [10]. Moreover, their abundancy in the genome correlates with the biological complexity [11]. NcRNAs are basically divided into highly expressed ‘housekeeping’ ncRNAs (ribosomal RNA, transfer RNA, small nuclear RNA, and small nucleolar RNA) and low-expressed and less abundant regulatory ncRNAs [12]. The latter group was described relatively recently and are of a particular interest given their functional role. Depending on the transcript length, they are further classified into short ncRNAs (20–200 nt) and long ncRNAs (>200 nt) [8,13].

Their multiplicity, localization in transcriptionally active parts of the genome, and deregulation in many types of cancer, including GC, were confirmed by a number of studies and make them valuable molecular candidates for diagnostics and therapeutic approaches [14,15].

This review addresses the role of E-cadherin and ncRNA-mediated play mechanisms of its regulation in the epithelial–mesenchymal transition (EMT) during stomach carcinogenesis. 

## 2. E-cadherin and Its Biological Significance

E-cadherin belongs to the family of type I classical cadherins. Its main function in the cell is to form the structures that establish and maintain cell–cell interactions. The protein was initially discovered in blastomeres of mouse embryos. Knockout E-cadherin is lethal in mice because mutant embryos fail to produce epithelium and to form organs and tissues essential for the development of a multicellular body [16].

E-cadherin is a transmembrane glycoprotein. Its extracellular domain harbors five repetitive motifs with calcium-binding sites between them. The calcium-binding motifs allow homophilic interactions with similar molecules of neighbor cells and the interactions link the cadherin molecules together, thereby ensuring tight cell–cell contacts [17]. The intracellular cytoplasmic domain is utilized by E-cadherin to bind with catenin family proteins. Binding with β- and p120-catenins yields a multiprotein complex, which interacts through α-catenin to actin filaments in the cell. A clustering of cadherin–catenin complexes on the cell membrane results in a local remodeling of intracellular actin and microtubules, thus facilitating the formation of cell–cell adherens junctions (AJs). AJs harbor actin regulatory proteins, such as the Arp2/3 complex, members of the Ena/VASP family, and the Wiskott–Aldrich syndrome protein (WASP). Their dysfunction leads to AJ destruction [18].

The cadherin concentration on the cell surface can change as a result of endocytosis, which mediates internalization of single unbound protein molecules into the cytoplasm. As cell–cell AJs form from actin-bound clustered cadherin, the cadherin molecules are stabilized on the cell surface and their endocytosis is inhibited [18].

## 3. Role of E-cadherin in Carcinogenesis: The Epithelial–Mesenchymal Transition (EMT)

With the decrease of E-cadherin abundance on the cell membrane due to downregulation of the gene, cell–cell interactions are attenuated or abolished. Loss of E-cadherin expression was earlier considered as a main cause of the EMT [19]. 

The EMT is characterized by suppression of the epithelial cell properties and behavior with concomitant and activation of the mesenchymal traits. This allows epithelial and endothelial cells to acquire a mesenchymal phenotype. The process is reversible at its early stage and may change to the mesenchymal–epithelial transition (MET), whereby cells return to the original epithelial phenotype. The expression of certain genes, including those for cadherins and integrins, is reprogrammed in the EMT. The EMT is thought to provide one of the main mechanisms that determine invasive and metastatic cancer cell spreading [20]. 

In the course of the EMT, cell contacts are destabilized in a coordinated manner via regulatory and epigenetic mechanisms. Additionally, the polar orientation of cells is impaired, the actin cytoskeleton is rearranged, and the cells consequently acquire a more mobile and invasive mesenchymal-like phenotype [7]. 

Current data demonstrate that loss of E-cadherin expression is a consequence rather than a cause of the EMT. Cultured *CDH1*-deficient cells did not display higher expression of mesenchymal markers and higher invasive properties. At the same time, the cells exhibited changes in the normal organization of microtubules and the actin cytoskeleton as well as substantial upregulation of the metalloproteinase genes *MMP9, MMP14, MMP15, MMP17,* and *MMP28*. Additionally, genes of cell adhesion molecules were downregulated, including the integrins ITGA1, ITGA4, ITGA5, ITGAV, ITGB1, and ITGB2 and their subunits α1β1, α2β1, α3β1, α4β1, α5β1, αvβ1, and α1β2, respectively [21].

A partial or complete loss of E-cadherin expression in the EMT is presumably mediated by mechanisms that block expression of E-cadherin regulators and the E-cadherin-associated transcription factors SNAIL1/2, ZEB, TWIST1, GRHL2, OVOL1/2, and PRRX1 [22]. The *CDH1* promoter harbors two conserved E-boxes, which bind with the majority of E-cadherin repressors, such as SNAIL1, SNAIL2/SLUG, TWIST, ZEB1, and ZEB2. The repressors exert an inactivating effect on *CDH1*, thus facilitating the EMT and promoting the survival and resistance of cancer cells [23]. The factors that prevent cadherin inactivation include the KLF4 transcription factor, which competes with ZEB2 for binding to the *CDH1* promoter to prevent its inactivation, while ZEB2 is bound by FOXA2 [24].

In the study, Yongju Xue et al. carried out the analysis of ZEB1 expression in GC tumor tissue and adjacent non-tumor tissues of the stomach. It was shown that the level of ZEB1 expression correlates with the degree of differentiation, metastasis to lymph nodes, and the stage of GC, indicating the important role of ZEB1 in the development and progression of GC. Inhibition of ZEB1 expression resulted in lower proliferation and migration of GC cells, and induction of apoptosis. They have also demonstrated that ncRNA ZEB1-AS1 is highly expressed, which regulates the expression of ZEB1, and ZEB1 can affect E-cadherin through specific binding. That leads to morphological changes in polarized epithelial cells underlying the impaired polarity that is needed for transformation into mesenchymal cells. The latter then move freely among the cell matrix, contributing to EMT. Suppression of ZEB1 may improve the expression of E-cadherin in GC cells and reduce the expression of vimentin, which indicates the participation of ZEB1 in the EMT initiation [25].

The cancer cell microenvironment can also regulate E-cadherin expression, acting through hypoxia-inducible factor 1 (HIF-1), PPAR-γ, and a higher affinity of the transcriptional activators GRHL3 and HNF4A to the *CDH1* enhancer regions, especially in secondary metastases [26].

While the current mechanistic understanding of the role of E-cadherin in carcinogenesis is far from being complete, E-cadherin is undoubtedly a substantial factor of invasion and metastasis. A tumor progression model that was proposed and accepted for breast cancer suggests that plasticity for E-cadherin changes, including both downregulation and re-expression. As the tumor develops, epithelial cells initially lose E-cadherin expression and become less differentiated and more invasive. The changes allow transformed cells to detach from the epithelial layer and to intravasate to the circulation. During a colonization of a secondary organ for a metastasis, disseminated cancer cells start expressing E-cadherin again. Its re-expression arises probably because metastatic cells need contacts with the target organ to allow a physical fixation of the metastasis and to prevent anoikis, which is cell death due to improper or lost cell adhesion (attachment to the extracellular matrix). Thus, E-cadherin re-expression, which is accompanied by changes in the expression of several other genes, is necessary for a successful generation of secondary metastases [27].

It is noteworthy that the EMT is not restricted to cancer cells, but is normally essential for embryogenesis, organ development, and wound healing. Collective movements of cell layers require cell–cell contacts to be preserved and are employed in normal morphogenesis in the course of embryo development [28]. 

## 4. Signaling Pathways Regulated by E-cadherin

E-cadherin is involved in several signaling pathways, such as the Wnt/β-catenin, Rho GTPase, and EGF/EGFR pathways, which are activated in carcinogenesis and play a role in many cancers, including GC [29,30].

In the normal cell, E-cadherin binds the catenin family proteins β-catenin and p120-catenin to produce multiprotein complexes. When the membrane amount of E-cadherin decreases, its association with β-catenin is distorted and free β-catenin accumulates in the cytoplasm and is subsequently transferred into the cell nucleus. In the nucleus, β-catenin binds with TCF/LEF1 (T-cell factor/lymphoid enhancer-binding factor 1) family transcription factors, which are thus released from their complexes with histone deacetylase to activate the expression of Wnt target genes, including *CD44, c-MYC, CCND1*, and *MMP7* [31]. Activation of these genes stimulates cell proliferation and promotes tumor progression. Normal E-cadherin expression prevents activation of the Wnt/β-catenin signaling pathway by sequestering β-catenin in cell–cell contact regions.

Infections with CagA-positive *H. pylori* are known to increase the risk of GC. CagA is a bacterial oncoprotein and plays a key role in *H. pylori*-induced GC. CagA facilitates dysregulation of the Wnt/β-catenin signaling pathway. It competitively interacts with E-cadherin to displace β-catenin, thereby preventing the formation of its complex and causing its cytoplasmic and nuclear accumulation, and inhibits β-catenin phosphorylation and proteasomal degradation in the cytoplasm [32].

Another pathway that involves E-cadherin, and is often distorted in GC, is associated with activation of the Rho GTPase cascade through RhoA, RAC1, and CDC42. These molecules play an important role in organizing the cytoskeleton, increase cell mobility, and are involved in acquiring the mesenchymal phenotype by the cell. In addition, upregulation of the Rho GTPase signaling pathway facilitates activation of proliferative signaling and dysregulation of the cell cycle [33]. 

Upregulation of RhoA, which enhances the migration properties of cells, was associated with missense mutations that affect the extracellular domain of E-cadherin and are characteristic of hereditary diffuse GC [34]. Activation of the Rho GTPase pathway can proceed through E-cadherin directly or through activation of the epidermal growth factor receptor (EGFR) [35]. Mutations of the E-cadherin extracellular domain may alter its interaction with EGFR, leading to EGFR activation and an additional increase in cell mobility through RhoA [36]. 

Moreover, loss of E-cadherin and the release of p12-catenin activates the RAC1-MAPK signaling pathway and promotes cell transformation and growth [37]. 

The E-cadherin/catenin complex is known to decrease the activity of nuclear factor kappa B (NF-κB) [38]. The signaling pathway that involves NF-κB regulates the epithelial cell phenotype during inflammation, which is associated with both carcinogenesis and *H. pylori* infection in the stomach [39].

NF-κB belongs to a group of transcription factors (RELA, RELB, c-REL, NF-κB1/P50, and NF-κB2/P52) that form homo- and hetero-dimers and increase or inhibit the expression of many genes. NF-κB can mediate activation of the cytokines/chemokines IL-1, IL-8, TNF, IL-6, and MCP-1; the pro- and anti-apoptotic factors cIAP, c-FLIP, A20, and BCL-XL; the vascular endothelial growth factor (VEGF); and matrix metalloproteinases 2 and 9 (MMP-2 and MMP-9) in normal and cancer cells in response to various stimuli. When activated, many of the molecules promote carcinogenesis in the stomach, thus making NF-κB signaling an interesting therapeutic target in GC patients [40]. 

In mammals, a canonical NF-κB activation pathway is mainly associated with p65:p50 dimers, which are stabilized in the cytoplasm via binding with IκB family proteins. Stimulation with a broad range of inflammatory mediators leads to their phosphorylation and subsequent degradation, and the p65:p50 dimers are consequently released from their complexes, transferred into the nucleus, and activate transcription of various target genes, including *BCL2, IL6,* and *TNF*. Activation of these targets improves cell viability, decreases apoptosis, and promotes carcinogenesis-associated inflammation [41].

Stimulation of E-cadherin expression was shown to inhibit the NF-κB pathway, whereas loss of E-cadherin expression activates NF-κB transcription [42]. However, recent experiments with EC96 GC cells, which lack E-cadherin expression and have a high-level of free β-catenin, showed that E-cadherin re-expression increased cell proliferation, although the activity of Wnt signaling was repressed. The analysis showed that NF-κB activation and the subsequent *c-MYC* induction in response to E-cadherin re-expression stimulate cell proliferation. To accelerate proliferation, EC96 cells increased glucose uptake and produced ATP via both mitochondrial oxidative phosphorylation and glycolysis, the processes being associated with NF-κB activation. Thus, E-cadherin re-expression and the consequent induction of NF-κB signaling likely stimulate energy metabolism and cell proliferation [43].

The functions of E-cadherin are broadly considered now, and its role is not restricted to tumor suppression. Abnormal regulation of E-cadherin distorts the related signaling pathways, thereby changing the cell polarity, altering the cytoskeleton organization, increasing the cell viability, activating the EMT, and eventually facilitating cell invasion and migration [44]. All of these events may act synergistically or separately to promote carcinogenesis, including that in the stomach.

## 5. Mechanisms of E-cadherin Inactivation in the Tumors

In GC cells, both genetic and epigenetic factors may affect the function of *CDH1*. In the case of genetic inactivation of the gene, its DNA is structurally damaged. Structural genetic inactivation includes *CDH1* mutations and loss of heterozygosity (LOH) as a result of a deletion. Genetic inactivation was observed in various sporadic tumors, whereas germline mutations are associated with hereditary early diffuse GC and hereditary lobular breast carcinomas [45]. 

CDH1/E-cadherin is involved in multiple processes in tissues, and its dysfunction can lead to various clinical manifestations. It is now widely known that in addition to diffuse gastric cancer (DGC) and lobular breast cancer (LBC), congenital malformations such as cleft lip/palate and BCDS (blepharocheilodontic syndrome) are a part of the spectrum of diseases associated with CDH1 deregulation. Thus, CDH1 can be characterized as a pleiotropic gene [46]. Pleiotropy is a phenomenon in which a defect of one gene can lead to various clinical effects. According to the GWAS (Genome-Wide Association Studies) catalog, 16.9% of genes and 4.6% of genetic variants have pleiotropic effects [47]. Although the emergence of pleiotropy is central in evolution, the mechanisms by which a single gene can affect multiple features are still incompletely understood.

It could be suggested that CDH1 pleiotropy is associated with the effects of each mutation. Due to specific mutations, E-cadherin may interact with proteins in different manners, activating distinct signaling pathways and causing a different cellular response. Besides, genetic background could also participate in the development of such an effect [48].

However, somatic and germline mutations of the coding region account for only a minor portion of cases with cadherin expression inactivated in cancer cells. Deletions and epigenetic alterations are the most common factors of the loss of function, including promoter methylation, histone modification, and noncoding RNAs. These mechanisms change the chromatin conformation and thus limit the access of cis-regulatory transcription factors to regulatory gene regions.

DNA methylation is an addition of the methyl group at C5 of the cytosine ring to produce 5-methylcytosine. Methylation of CpG islands in the promoters of tumor suppressor genes leads to their inactivation by preventing access of transcription factors to their binding sites in the promoter and thus blocking transcription [49]. Promoter hypermethylation was identified as one of the main mechanisms that inactivates *CDH1* during the progression of various cancers, including GC [50]. Interestingly, external etiological factors, such as the hepatitis C and Epstein–Barr viruses, are also capable of inducing *CDH1* promoter hypermethylation [51,52]. It is not surprising that Epstein–Barr virus-associated GC, which is a GC subtype identified in The Cancer Genome Atlas (TCGA) whole-genome studies, is characterized by a certain pattern of promoter hypermethylation in specific genes, such as *CDH1, P15, P16/INK4a,* and *P73* [7,53].

Hypermethylation of various genes in gastric tissues is currently associated with a higher risk of tumorigenesis. Hypermethylation of *CDH1* and other genes was observed in nontumor mucosa samples from GC patients [54,55].

It is of interest that inflammation-inducing *H. pylori* infection modulates the promoter methylation status of tumor suppressor genes, including *CDH1,* during GC development and progression [56]. 

Apart from promoter hypermethylation, various posttranslational changes may epigenetically inactivate cadherin expression. Such changes include covalent modifications (methylation, acetylation, phosphorylation, and SUMOylation) of the protruding N- and C-terminal tails of histones. The modifications regulate the state of chromatin, either causing its condensation into heterochromatin and thereby activating the gene, or leading to de-condensation into euchromatin and thus inactivating gene expression [57,58]. For example, upregulation of SNAIL, which acts as an E-cadherin repressor, facilitates methylation of histone H3 at Lys9 (H3K9me3) in the promoter of the E-cadherin gene, and thus inhibits its expression [59]. In addition, ZEB1 and ZEB2, which also act as E-cadherin repressors, were shown to activate histone deacetylase (HDAC) leading to histone deacetylation, the formation of an inactive dense chromatin conformation, and eventually, a decrease in cadherin expression [60].

A growing body of data indicate that noncoding RNAs, such as microRNAs (miRNAs) and long noncoding RNAs (lncRNAs), play a critical role in carcinogenesis [61,62,63].

Both lncRNAs and miRNAs act alone or together to regulate various biological processes, including the immune response, apoptosis, cell growth and differentiation, metastasis, and drug resistance [64,65,66].

**Figure 1:**
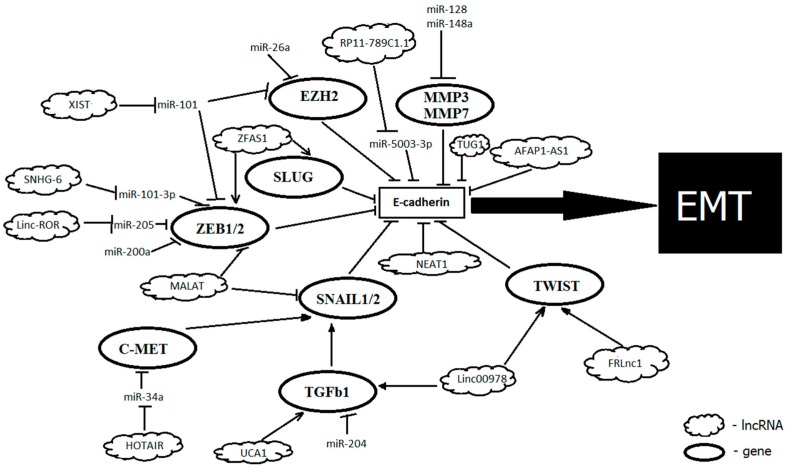
Noncoding RNAs that facilitate the epithelial–mesenchymal transition (EMT) in Gastric cancer (GC) by acting on E-cadherin.

Several noncoding RNAs, shown to play a substantial role in the epigenetic control of gene expression in carcinogenesis, regulate the E-cadherin function, acting directly or through numerous factors that regulate transcription of its gene, such as SNAIL1, ZEB1, and ZEB2 [67] (Figure 1).

## 6. Role of MiRNAs in Regulation of E-cadherin Expression in Gastric Cancer

MiRNAs are the most extensively investigated group of short noncoding transcripts of 19–25 nt that regulate gene expression post-transcriptionally by suppressing translation or inducing degradation of target mRNAs. The mechanism depends on the extent to which 5′-terminal region 2–8 of a miRNA is complementary to the 3′-UTR of its target mRNA. A perfect complementarity leads to mRNA degradation via RNA interference, whereas 2 or 3 mismatches result in the suppression of target mRNA translation [68]. A total of 2654 miRNAs have been described to date for the human genome [69]. Because miRNAs are multiple, occur in all cells, and are each capable of affecting the expression of several dozens of target genes, they are an important component of genome regulation and play an important role in various biological processes [70].

The miRNAs that act as positive regulators of E-cadherin support epithelial cells by preventing the EMT and suppressing invasion. For example, miR-26a prevents the EMT and facilitates E-cadherin activation by inactivating the Polycomb repressive complex 2 (PRC2) subunit EZH2, which trimethylates Lys27 in histone H3 (H3K27) and induces the EMT during carcinogenesis [71]. Alternatively, the miRNAs that negatively regulate E-cadherin promote the invasive and tumorigenic properties of cells. Specifically, miR-200c is downregulated in GC and represses E-cadherin through targeting ZEB1, which leads to poorly differentiated histology in GC cells [72]. Lack of miR-101 distorts the E-cadherin function by regulating EZH2 in intestinal GC [73]. However, a miRNA may act differently in various tumors. Upregulation of miR-9 represses E-cadherin expression in squamous cell carcinoma of the esophagus, leading to β-catenin transfer and initiating the EMT [74]. The same miR-9 blocks the E-cadherin repressor SNAIL1 and upregulates E-cadherin expression through NF-κB1 during melanoma progression [75].

A growing body of knowledge now supports the finding that generation of an extracellular fragment of E-cadherin via its cleavage by proteolytic enzymes plays an important role in tumor invasion and metastasis. Recent studies showed that *H. pylori* is capable of activating matrix metallopeptidases (MMPs) in GC cells, thus stimulating the enzymatic cleavage of E-cadherin [76]. It was observed that *H. pylori* substantially decreases miR-128 and miR-148a expression in cultured SGC-7901 GC cells to stimulate expression of MMP-3 and MMP-7 and to increase their effect on E-cadherin proteolysis, thereby inducing cell migration and invasion. Activation of the MMP/E-cadherin pathway in human GC cells infected with *H. pylori* is associated with the miR-128/miR-148a regulation [77]. Besides, miR-148a expression suppresses cell invasion and migration in GC by regulating DNMT1 expression [78]. Finally, the *SMAD2* gene was also identified as the direct and functional target of miR-148a, which could be a mechanism of the EMT regulation [79]. Another miRNA, miR-29b/c, which is significantly correlated with the degree of differentiation and invasion of the GC cells, was determined as directly targeting DNMT3A. Its deregulation leads to the epigenetic silencing of CDH1 and contributes to the metastasis phenotype in GC [80]. 

MiRNAs can affect E-cadherin expression by direct targeting of *CDH1* or regulating it indirectly via genes from signaling pathways. Expression of miR-204 in malignant gastric tumors, adjacent normal tissues, and GC cell lines was found to be far lower than in normal epithelial cells. Upregulation of miR-204 suppresses GC cell proliferation, invasion, and migration through the TGF-β signaling pathway, upregulates E-cadherin expression, and considerably downregulates expression of N-cadherin, vimentin, fibronectin, SNAIL, and TWIST. It was found that miR-204 directly targets TGFBR2, which is expressed to a high level in GC and acts as an upstream regulator of TGF-β, which modulates the EMT [81]. MiRNAs described to directly target *CDH1* (Table 1) are less numerous. A study by Chen et al. showed that miR-5003-3p promotes migration, invasion, and the EMT in GC by directly targeting the 3′ UTR of *CDH1* at sites A and B [82]. MiR-217 also repressed the expression of CDH1 3′UTR. MiR-217 overexpression enhanced gastric cancer cells proliferation and reduced the exosomal level of CDH1, which can be delivered into the microenvironment [83]. Using LNA anti-miRNA oligonucleotides with a complementary sequence to miRNA-9, Lima et al. demonstrated that miRNA-9, which is upregulated in GC, could not only affect E-cadherin expression indirectly, but also targeted *CDH1* 3′UTR, thereby triggering cell motility and invasiveness [84]. Yanaka et al. demonstrated that overexpression of miR-544a reduced the expression of its direct targets CDH1 and AXIN2, which not only led to EMT, but also subsequently induced the nuclear import of β-catenin and activation of the WNT signaling pathway. That suggests miR-544a as a potential prognostic marker and therapeutic target for metastatic GC [85]. 

### MiRNAs in Diagnostic and Therapy of Gastric Cancer 

Because of the important role in EMT regulation, almost all aforementioned miRNAs are potentially of diagnostic and therapeutic value. There are already several drugs that have been clinically approved for serious disorders, including cancers, and a number of diagnostic panels. However, none of them have been applied in GC yet. Among the most prominent miRNA candidates, involved in EMT and E-cadherin regulation is miR-376a, which is associated with advanced disease and poor prognosis in GC patients [88]. MiR-381 is another potential target for GC therapy [89], which acts as a tumor suppressor in GC by directly affecting TMEM16A and regulating the TGF-β signaling pathway and EMT.

Recent studies additionally confirmed that the EMT and therefore, miRNAs involved in its regulation, play a role in drug resistance [144]. For example, miR-128 is downregulated in paclitaxel-resistant GC cells [145]. Promising results for overcoming drug resistance were obtained with the miRNAs that upregulate E-cadherin expression, because this upregulation reverts the EMT, decreases the invasive properties of cancer cells, and activates proapoptotic pathways [27].

## 7. Long Noncoding RNAs Involved in the EMT and Regulation of E-cadherin Expression in Gastric Cancer

The lncRNAs is the most numerous class of ncRNAs which includes transcripts of more than 200 nt with a limited or no protein-coding capacity. The lncRNA length is usually far greater than 200 nt, and for 2482 humans, lncRNAs were determined to be longer than 1000 nt [12].

LncRNAs are associated with many physiological and pathological processes, such as cell growth, differentiation, development, and carcinogenesis [146,147,148]. Abnormal lncRNA expression affects not only cancer cell proliferation and metastasis, but also the EMT, tumor drug resistance, and cancer stem cells [149,150]. Several studies showed that lncRNAs are capable of facilitating tumor progression and metastasis in various cancers not only by directly regulating gene expression, but also by acting as competing endogenous RNAs (ceRNAs), which function as sponges for miRNAs and act to regulate the downstream genes and to play an important role in physiological and pathological processes [151,152]. 

Many lncRNAs are currently known to play various roles in gastric carcinogenesis and to provide favorable or unfavorable GC markers, the latter being indicative of disease progression [153]. Similar to protein-coding genes, lncRNAs are classified as oncogenic or tumor suppressors depending on their role in carcinogenesis.

### 7.1. Oncogenic ncRNAs in Gastric Cancer 

HOX Antisense Intergenic RNA (HOTAIR) was among the first discovered lncRNAs and still remains one of the most extensively investigated. HOTAIR is upregulated in many cancers, including GC, where HOTAIR increases the EMT and thereby facilitates metastasis. Expression of E-cadherin is elevated in HOTAIR knockdown cells compared with HOTAIR-overexpressing cells. This mechanism is thought to involve HOTAIR recruitment and Polycomb-repressive complex 2 (PRC2) binding with epigenetic inactivation of miR-34a, which activates the HGF/c-MET/SNAIL pathway and thereby facilitates the EMT in cancer cells [109]. Another mechanism by which HOTAIR stimulates EMT is associated with switching the acetylation of histone H3 lysine 27 to the methylation of the E-cadherin promoter, which induces transcriptional inhibition of E-cadherin. HOTAIR recruits a PRC2 for H3K27me3 catalysis. The loss of PRC2 activity as the result of HOTAIR knockdown can lead to a global decrease of H3K27 methylation and an increase of H3K27 acetylation. The results of the study performed by Song et al. suggest that the HOTAIR-mediated switch from acetylation to methylation was associated with the inhibition of E-cadherin transcription by epigenetic mechanisms, the E-cadherin promoter was switched from a transcriptionally active state to transcriptionally repressive, thus contributing to the development of GC [110].

Certain transcription factors are known to inhibit E-cadherin expression, as is the case with SNAIL, SLUG, and ZEB1 [138]. Several lncRNAs affect those factors, thus regulating E-cadherin in an indirect manner. For example, lncRNA XLOC_010235 is expressed to a high level in GC tissues and is capable of inactivating the EMT inducer SNAIL1, thereby regulating the EMT and disease progression [113]. XLOC-010235 hyperexpression is also accompanied by an increase in the expression of N-cadherin and vimentin, an increase in the amount of MMP2/MMP9, and a substantial decrease in E-cadherin expression [113,118]. The lncRNA ZFAS1 (ZNFX1 antisense RNA1) is expressed to higher levels in GC tissues, the serum, and exosomes. ZFAS1 activates the EMT inducer ZEB1 and ensures ZEB2 stability upon activation of EMT signaling. Exosomes originating from GC cells may promote distant metastasis by transferring ZFAS1 [114]. A ZFAS1 knockdown downregulates the expression of MMP2, MMP9, N-cadherin, β1-integrin, ZEB1, TWIST, and SNAIL, while substantially upregulating E-cadherin expression [115].

MALAT1 (metastasis-associated lung adenocarcinoma transcript 1) is another well-known example of oncogenic lncRNA. Chen et al. showed that inhibition of its expression occurs in GC cells and is accompanied by an increase in E-cadherin expression [116]. UPF1 was found to negatively correlate with MALAT1 expression, while its upregulation inhibits GC cell migration, invasion, and EMT [154]. In contrast, elevated MALAT1 expression in GC cells decreases the effects of UPF1, including its capabilities of suppressing cell proliferation and the EMT, and increasing apoptosis [62]. Thus, UPF1 directly binds and downregulates MALAT1 to inhibit GC progression. More recent studies have confirmed that MALAT1 regulates the expression of SNAIL, N-cadherin, and ZEB1 and acts through these factors on the EMT [117].

It was identified that novel lncRNA FRLnc1 is expressed rather highly in GC cell lines. A functional analysis in vitro and a model of lung cancer metastasis showed that FRLnc1 improves the capability of migration in cancer cells. It was found that FRLnc1 is regulated by oncogenic FOXM1 and acts as an EMT promoter by activating the TGFβ-1 and TWIST downstream genes to affect GC cell migration.

The lncRNA LINC00978 level is greatly increased in the plasma and tissues of GC patients. A LINC00978 knockdown upregulates p21 expression, increases apoptosis of GC cells, and decreases their migratory and invasive functions. In addition, the knockdown prevents the expression of TWIST1 and SLUG (SNAIL2) and inhibits N-cadherin and vimentin, while considerably upregulating E-cadherin expression. These features characterize LINC00978 as an EMT inducer. A LINC00978 knockdown downregulates TGF-β expression and inhibits SMAD2 activation and MMP9 expression in GC cells. LINC00978 may induce the EMT by activating the TGF-β/SMAD regulatory pathway [119].

The lncRNA UCA1 (urothelial carcinoma-associated 1) is involved in the development and progression of many cancers, including GC. UCA1 upregulation that was induced in GC cells with TGFβ-1 was associated with the depth of invasion, metastasis to lymph nodes, and distant metastasis. UCA1 inactivation in GC cells decreased the levels of vimentin and SNAIL, which are associated with the EMT, and increased the levels of E-cadherin and ZO-1 (zonula occludens-1). The UCA1 effect was possible to restore in part with the use of TGFβ1 [120].

The lncRNA TUG1 (taurine upregulated gene 1) is differently expressed in different tissues and performs oncogenic or tumor suppressor functions in different cancers. Its expression is substantially elevated in GC tissues, and its knockdown suppresses GC cell proliferation both in vitro and in vivo. TUG1 occurs predominantly in the nucleus, interacts with PRC2, and regulates gene expression at the transcriptional level. The interaction with PRC2 is necessary for the epigenetic repression of cyclin-dependent kinase inhibitors, including P15, P16, P21, and P57, thus negatively regulating the cell cycle and increasing GC cell proliferation [121]. TUG1 upregulation in colorectal cancer is accompanied by downregulation of E-cadherin, as well as upregulation of N-cadherin and vimentin, suggesting a possible regulation of the EMT [122].

As another common mechanism of gene regulation, lncRNAs interact with miRNAs, thereby modulating its availability to endogenous mRNA targets. Being localized in cytoplasm and containing miRNA recognition elements for one or multiple miRNAs in their sequence, lncRNAs can bind them, preventing its interaction with target genes, and thereby regulate them indirectly [155]. According to this mechanism, lncRNAs often regulate E-cadherin and EMT by downregulating miRNAs and thus upregulating their target genes. Apart from the already mentioned HOTAIR, this mechanism was described for the oncogenic lncRNA, Linc00152, which directly inhibits the expression of miR-193b-3p and thus abolishes its anti-proliferative, anti-migration, and anti-invasive effects in GC, leading additionally to ETS1 upregulation [123]. LncRNA XIST is another example, which is expressed from the inactive X chromosome, acts as a sponge for miR-101, and modulates EZH2 in GC cells [124]. 

Saito et al. showed that lncRNA activated by TGF-β (lncRNA-ATB) is upregulated in GC cells and is capable of simultaneously inducing ZEB1 expression and inhibiting miR-200, thus leading to the EMT in cancer cells. Reduction of lncRNA-ATB expression induced E-cadherin and repressed the mesenchymal markers ZEB1 and N-cadherin. Thus, lncRNA-ATB plays a role in the EMT in GC through the TGF-β/miR-200/ZEB regulatory axis [125].

The lncRNA SNHG6 is upregulated in GC tissues and cell lines, which is accompanied by deep invasion and metastasis to lymph nodes and distant tissues. Its inactivation alleviates the malignant process and decelerates the EMT. It was shown that SNHG6 may epigenetically inactivate P27 through EZH2-dependent histone H3 methylation (H3K27me3) in the promoter of the *P27*. SNHG6 promotes the EMT by acting as a ceRNA, or a molecular sponge for miR-101-3p, thereby upregulating ZEB1 at the post-transcriptional level and enhancing cancer cell migration [64]. In addition, SNHG6 inactivation was observed not only to decrease the EZH2 expression level, but also to activate the JNK (c-JUN N-terminal kinase) pathway and to upregulate P21 [127].

### 7.2. Tumour Suppressor ncRNAs in Gastric Cancer 

The lncRNAs that are capable of inhibiting GC progression are classified as tumor suppressor lncRNAs. For example, the lncRNA AF147447 inhibits proliferation and invasion of GC cells, directly binding to the promoter region of MUC2, which possesses oncogenic properties. In addition, AF147447 may regulate the expression of miR-34c, which targets MUC2, EGFR, and CD44. Upregulation of AF147447 induces miR-34c and substantially represses MUC2 and EGFR. Thus, AF147447 can post-transcriptionally alter MUC2 expression by binding with miR-34 [128].

Zhao et al. observed that lncRNA SNHG5 expression is substantially decreased in GC cells and tissues, while MTA2 (metastasis-associated protein 2) expression is increased. MTA2 possesses oncogenic properties. It is a component of the NuRD complex, which regulates nucleosome remodeling and histone deacetylation and plays a role in the transcriptional regulation of various signaling pathway components, including E-cadherin, P21, Ki-67, and KAI-1, which are involved in regulating cell growth and metastasis. Inactivation of MTA2 expression restores E-cadherin expression in GC cells with a SNHG5 knockdown. High-level SNHG5 expression may provide a cytoplasmic trap for MTA2 because SNHG5 can directly bind MTA2 to prevent its transfer from the cytoplasm into the nucleus, thus inhibiting the MTA2 functions in GC cell growth and metastasis [129]. 

The lncRNA Linc00261 almost lacks expression in GC cells and correlates with deep invasion, metastasis, and poor prognosis. This is accompanied by low-level expression of E-cadherin and high-level expression of N-cadherin, FN1, and vimentin, corresponding to the EMT in GC cells. SLUG is one of the key EMT mediators, and its expression is associated with a decrease in E-cadherin and an aggressive GC phenotype. Linc00261 is capable of binding with SLUG to impair its stability and facilitates its degradation by increasing the interaction of SLUG with GSK3b [130].

### 7.3. LncRNAs in Diagnostic and Therapy of Gastric Cancer 

Thus, many lncRNAs are differentially expressed in normal and GC cells, and some of them correlate with different clinical courses of the disease. Moreover, changes in lncRNA expression in primary tumors are reflected in the lncRNA contents in biological fluids. That means that respective lncRNAs could be considered as potential markers for non-invasive diagnostic [156].

The lncRNA SPRY4-IT1 is a potential prognostic marker, its gene is in an intron of the Sprouty 4 protein-coding gene (*SPRY4*) [157]. SPRY4-IT1 is localized in the cytoplasm, has several long hairpins in its secondary structure, can function as a molecular scaffold for protein complexes that lack protein–protein interaction domains, and can directly interact with miRNAs to prevent their binding with mRNAs, thereby regulating protein synthesis. A SPRY4-IT1 knockdown inhibited cell growth and differentiation and induced apoptosis in melanoma cells [157]. SPRY4-TI1 was found to stimulate cell proliferation, migration, and invasion by regulating the EMT in various cancers, including GC, colorectal cancer, etc. [158,159]. Its upregulation is associated with poor prognosis in GC [160,161].

The lncRNA AFAP1-AS1 (AFAP1 antisense transcript 1) was discovered recently. Its substantial upregulation was observed in GC tissues and cell lines and is associated with invasion in lymph nodes, distant metastasis, advanced TNM stages, and poor prognosis. Inactivation of AFAP1-AS1 in GC cell lines was shown to suppress cell proliferation and invasion in vitro and to induce apoptosis by decreasing the BCL-2 level and increasing the levels of PARP (poly(ADP-ribose) polymerase), caspase 3, caspase 9, PTEN, and BAX. An AFAP1-AS1 knockdown affects the EMT by upregulating E-cadherin and downregulating N-cadherin and vimentin [131,132].

Upregulation of the lncRNA CASC15 is similarly associated with poor prognosis in cancer patients. CASC15 inactivation leads to E-cadherin expression and downregulates N-cadherin expression, thus regulating the EMT. CASC15 interacts with EZH2 and WDR5, recruits them to the *CDKN1A* promoter region, and thus modulates *CDKN1A* expression in the nucleus to play its role in gastric carcinogenesis. In addition, CASC15 acts as a sponge for miR-33a-5p and activates ZEB1 in the cytoplasm [133].

High-level expression of the lncRNA ZEB1-AS1 in GC cell lines and tissues correlates with lymph node metastasis, TNM stage, and poor overall survival of patients. Inactivation of ZEB1-AS1 decreases GC cell proliferation and invasion in vitro [134]. In addition, ZEB1-AS1 downregulates miR-335-5p expression by acting as a molecular sponge [135]. 

The lncRNA NEAT1 (nuclear-enriched abundant transcript 1) is hyper-expressed in GC tissues and cell lines and correlates with more advanced stages, lymph node and distant metastasis, and a low overall survival in patients. Inactivation of NEAT1 expression substantially inhibits GC cell migration and invasion in vitro, downregulates mesenchymal markers, including vimentin and N-cadherin, and upregulates epithelial markers, such as ZO-1 and E-cadherin. However, its effect does not involve MMP-2 and MMP-9. Thus, NEAT1 regulates GC cell mobility through the EMT [136]. Additionally, it was observed that NEAT1 upregulation is accompanied by high-level expression of STAT3 and inactivation of miR-506, suggesting a role in GC for the NEAT1/miR-506/STAT3 regulatory axis [137].

The suppressor lncRNA RP11-789C1.1 is downregulated in GC tissues compared with adjacent normal tissues and is far lower in lymph nodes with GC metastases than in metastasis-free lymph nodes. Patients with inactive RP11-789C1.1 displayed a far lower survival rate [138]. Chen et al. studied the effect of RP11-789C1.1 on the GC cell phenotype and found that abnormal expression of RP11-789C1.1 can substantially regulate the expression of many EMT-associated proteins, including E-cadherin, N-cadherin, and vimentin. The regulation proceeds via a direct binding of miR-5003-3p, which facilitates the malignant process by downregulating E-cadherin. Thus, RP11-789C1.1 affects GC cell invasion and metastasis through the miR-5003–EMT regulatory association [82].

Jia et al. recently demonstrated on the GC cell lines that the HOTAIR/miR-17-5p/PTEN axis significantly facilitates the viability, EMT process, and proliferation of GC cells that were subject to treatment of chemo-therapies and suggests them as potential targets for GC treatment [111].

A therapeutic potential was additionally reported for some of the lncRNAs studied in GC. For example, the lncRNA LINC00675 is expressed in GC tissues to a far lower level than in adjacent noncancerous tissues, and its low expression is associated with poor survival. LINC00675 was found to suppress GC cell proliferation, migration, and invasion in vitro and to inhibit distant metastasis to the lungs and liver in vivo. LINC00675 interacts with vimentin, which is one of the main EMT regulators, and increases the level of its phosphorylation at Ser83, thereby stimulating vimentin degradation and decreasing metastasis of GC cells. Thus, the LINC00675/vimentin complex may provide a potential therapeutic target in GC [139]. Han et al. identified LEIGC, a new differentially expressed lncRNA. Its overexpression may inhibit the EMT by downregulating vimentin, SNAIL, SLUG, ZEB, and TWIST and upregulating E-cadherin. In addition, high-level LEIGC expression increases the 5-fluorouracil sensitivity in GC cells [162]. 

## 8. Conclusions

The analysis of a large body of data from many studies shows that E-cadherin is now thought to have a far greater range of functions than merely sustaining cell adhesion in order to maintain the integrity of the epithelial cell layer. E-cadherin is involved in regulating proliferative signaling, cell differentiation, apoptotic properties, and migration. E-cadherin acts as a tumor suppressor protein and is necessary for regulating the cell functions whose distortion often leads to carcinogenesis. In GC, the loss of E-cadherin expression stimulates cell transformation into a more invasive and less differentiated state through the EMT process. 

Testing for E-cadherin expression and abnormalities of *CDH1* is promising for clinical diagnosis, prognosis, and therapy in GC. Because aberrant E-cadherin expression is associated with cancer initiation and progression, its therapeutic regulation via epigenetic mechanisms, such as methylation/demethylation of the promoter gene region or use non-coding transcripts, such as miRNAs and lncRNAs, may provide a promising strategy in the future. Furthermore, being the regulators of the E-cadherin and EMT, ncRNAs itself could be used as therapeutic targets in GC and aberrations of their expression in the cells is a promising prognostic and diagnostic marker.

However, several questions remain open. First is whether E-cadherin is suitable as a biomarker on its own or a higher sensitivity and specificity of GC diagnosis and prognosis can be achieved by combining it with other key molecules. We think it is more efficient to use a set of molecular genetic markers (a diagnostic panel), including various lncRNAs that are specifically expressed in GC tissues. The second question is whether interventions targeting E-cadherin should be performed upon its downregulation or loss. Recent studies indicate that E-cadherin re-expression can be achieved via epigenetic mechanisms, for example, with the miR-200 family molecules that target the E-cadherin receptors ZEB1 and ZEB2 or affect *CDH1* expression. However, E-cadherin re-expression does not always exert an anti-tumor effect because malignant cells can use molecular bypasses to sustain the tumor growth. 

The current period is a period of data accumulation. Although ample, the knowledge that is available for new ncRNAs specifically expressed in various cancers, their regulatory interactions, and their effects on various signaling pathways fails to provide unequivocal answers as yet. The review focused on the role that ncRNAs play to upregulate or downregulate E-cadherin expression or to affect its regulators, thereby determining the EMT–MET pathway. The understanding of mechanisms and key players of this regulation could significantly update the current picture of cancer development. Moreover, these ncRNAs, both miRNAs and lncRNAs, may certainly provide good markers to predict the clinical course of GC, its aggressiveness, invasiveness, and metastatic potential, and to evaluate the efficacy of therapies. A diagnostic and prognostic potential is assumed for most of the deregulated ncRNAs considered in the review. Among the most prominent are miR-376a, miR-381, miR-128, HOTAIR, MALAT, CASC15 etc. As for their possible therapeutic potential, definite conclusions must wait until further studies experimentally evaluate all possible regulatory pathways and interactions, identify all targets, and estimate the safety of potential molecular genetic- and protein-based regulatory interventions. 

## Figures and Tables

**Table 1:** Non-coding (Nc)RNAs, participating in EMT regulation in GC.

NcRNA	Status in GC	Targets	Mechanism of Action	Functional Role	Reference
**microRNAs:**					
miR-5003-3p	Up	*CDH1*	directly targets the 3′UTR of *CDH1* at sites A and B	promotes migration, invasion and EMT	[82]
miR-200c	Down	*ZEB1*	targets *ZEB1* and thus increase E-cadherin expression	suppresses invasion and EMT	[72]
miR-101	Down	*EZH2*	targets *EZH2* and thus increase E-cadherin expression	suppresses EMT	[73]
miR-148a	Down	*SMAD2*	binds the 3′UTR of the *SMAD4* and suppresses TGFβ-induced EMT	suppresses EMT, cell invasion and migration; low expression associated with advanced clinical stage and poor prognosis in GC	[79]
miR-29b/c	Down	*DNMT3A*	targets *DNMT3A*, thus modulating methylation of *CDH1* promoter	suppresses EMT; significantly correlates with the degree of differentiation and invasion of the GC cells	[80]
miR-204	Down	*TGFBR2*	targets *TGFBR2*, regulating TGF-β	suppresses EMT, proliferation, invasion and migration	[81]
miR-217	Up/Down	*CDH1, PTPN14*	directly targets the 3′UTR of *CDH1*; directly targets the 3′UTR of *PTPN14*	promotes cells proliferation; suppresses EMT, low expression is correlated with metastasis	[83,86]
miRNA-9	Up/Down	*CDH1, RAB34, NFKB1, CDX-2*	targets 3′UTR of *CDH1*; being downregulated, target RAB34 and NFKB1, regulating E-cadherin indirectly	triggering cell motility and invasiveness, regulates EMT	[84]
miR-544a	Up	*CDH1*	directly targets *CDH1* and *AXIN2*, regulate WNT signaling pathway	promotes EMT, cell motility and invasion; potential therapeutic target for metastatic GC	[85]
miR-199a-5p	Up	*CDH1*	directly targets the 3′UTR of *CDH1*	promotes EMT, cell invasion and metastasis; potential therapeutic targets and biomarkers for GC progression	[87]
miR-376a	Down	n/a	n/a	associated with advanced GC and poor prognosis	[88]
miR-381	Down	*TMEM16A*	targets *TMEM16A*, thus regulating the TGF-β signaling pathway and EMT	suppresses EMT, decreases cell proliferation, migration and invasion	[89]
miRNA-96-5p	Down	*FoxQ1*	binds to the 3′UTR of *FoxQ1*, decreasing the protein level of FoxQ1; upregulates the expression of E-cadherin and downregulates the expression of vimentin	suppresses the proliferation, migration and EMT	[90]
miR-574-3p	Down	*ZEB1*	bounds 3′-UTR of *ZEB1*, thus upregulating E-cadherin expression, and concomitantly downregulating the expression of vimentin.	inhibits cancer cell migration, invasion, EMT; modulates cisplatin sensitivity in vitro and in vivo	[91]
miR-1254	Down	*SMURF1*	downregulating *SMURF1* and thus inhibits EMT and decreases the PI3K/AKT signaling pathway	inhibits proliferation, migration, invasion, and EMT	[92]
miR-588	Down	*EIF5A2*	directly binds to 3′-UTR of *EIF5A2*	suppresses cell invasion, migration, and progression of EMT	[93]
miR-218	Down	*BMI1*, *WASF3*	inhibits the expression of BMI1 and its downstream targets p-Akt473 and MMPs; directly inhibits expression of WASF3	inhibits EMT, proliferation, invasion, and migration	[94,95]
miR-370	Down	*PAQR4*	directly inhibits expression of PAQR4	inhibits the proliferation, invasion, and EMT	[96]
miR-711	Down	*CD44*	targets *CD44* and thus downregulates vimentin expression and upregulates E-cadherin expression	inhibits the invasion, migration, and EMT	[97]
miR-543	Up	*SPOP*	directly inhibits expression of SPOP	promotes EMT, cell migration, and invasion	[98]
miR-361-5p	Down	*FOXM1*	suppresses the expression of MMP-3, MMP-9 and VEGF, increases expression of E-cadherin; acting through Wnt/β-catenin pathway; targets *FOXM1*, acting through the PI3K/Akt/mTOR pathway	inhibits EMT, cell proliferation, and mobility; low expression is correlated with larger tumor size and advanced TNM stage.	[99,100]
miR-592	Up	*SPRY2*	targets SPRY2 and acting through PI3K/AKT and MAPK/ERK signaling pathways	promotes proliferation, migration, and invasion, induces the EMT	[101]
miR-616-3p	Up	*PTEN*	directly inhibits expression of PTEN	promotes EMT, angiogenesis and metastasis; high expression is correlated with poor prognosis	[102]
miR-495	Down	*TWIST1*	directly inhibits expression of TWIST1	decreases cell viability and migration, increases apoptosis and inhibits the EMT	[103]
miR-1271	Down	*FOXQ1*	directly suppressing FOXQ1 expression	suppressed cell proliferation, invasion, and EMT; correlated with tumor size, tumor stage, lymph node metastasis, and TNM stage	[104]
miR-491-5p	Down	*SNAIL*	directly inhibits SNAIL expression; indirectly inhibits FGFR4, also decreasing the SNAIL level	suppresses EMT and tumor metastasis	[105]
miR-338-3p	Down	*ZEB2* and *MACC1*	targets *ZEB2* and MACC1/Met/Akt signaling, thus upregulating the E-cadherin and downregulating the N-cadherin, fibronectin, and vimentin	inhibits EMT, migration, and invasion	[106]
miR-124	Down	*SNAIL2*	represses the SNAIL2 expression	inhibits EMT, cell proliferation, and invasion; lower expression is associated with tumor size, lymphatic metastasis, and TNM stage	[107]
miR-379	Down	*FAK*	directly binds to 3′-UTR of *FAK*, resulting in suppression of AKT signaling	inhibited cell migration, invasion and EMT; low expression is associated with poor prognosis, lymph node metastasis, and advanced TNM stage	[108]
**Long non-coding RNAs:**					
HOTAIR	Up	PCR2, miR-34a, c-MET, SNAIL1, CDH1, miR-152	switching the acetylation of histone H3 lysine 27 to the methylation of the E-cadherin promoter, inducing its transcriptional inhibition; inactivates miR-34a, which activates the HGF/c-MET/SNAIL pathway and thus indirectly inhibits E-cadherin; targets miR-17-5p and thus regulates expression of PTEN	promotes EMT, facilitates viability, proliferation, and metastasis; higher expression correlates with lymphatic metastasis and TNM stage	[109,110,111,112]
XLOC_010235	Up	SNAIL1	inactivates SNAIL1, thereby upregulating E-cadherin expression	promotes EMT; high expression correlates with metastasis and TNM stage	[113]
ZFAS1	Up	ZEB1	activates the EMT inducer ZEB1	promotes EMT	[114,115]
MALAT1	Up	SNAIL, N-cadherin, ZEB1	targets SNAIL, N-cadherin, and ZEB1, thus decreasing E-cadherin expression	promotes EMT, invasion, angiogenesis, and metastasis	[116,117]
FRLnc1	Up	TWIST, TGFβ-1	activates the TGFβ-1 and TWIST	promotes EMT, invasion, and migration of cells	[118]
LINC00978	Up	TGFβ/SMAD, TWIST, SLUG	activates the TGF-β/SMAD regulatory pathway, thus decreasing E-cadherin expression	promotes EMT, invasion, and migration of cells, decreases apoptosis	[119]
UCA1	Up	TGFβ	targets TGFβ, decreases the levels of vimentin and SNAIL, thus regulating levels of E-cadherin and ZO-1	promotes EMT, associated with invasion and metastasis	[120]
TUG1	Up	CDH1	interacts with PRC2, epigenetically repressing cyclin-dependent kinase inhibitors (P15, P16, P21, and P57); downregulation of E-cadherin	promotes EMT, cell proliferation, and metastases, predicts a poor prognosis	[121,122]
Linc00152	Up	miR-193b-3p	directly inhibits expression of miR-193b-3p, leading additionally to ETS1 upregulation	promotes EMT, proliferation, migration, and invasion	[123]
XIST	Up	miR-101	acts as a sponge for miR-101, and modulates EZH2 expression	promotes EMT, cell proliferation, and invasion	[124]
lncRNA-ATB	Up	miR-200	acts through the TGF-β/miR-200/ZEB regulatory axis, thus decreasing E-cadherin expression	promotes EMT	[125]
SNHG1	Up	miR-140	acting as a sponge, repress miR-140 expression and thereby elevated its down-stream target ADAM10	promotes EMT, proliferation, and invasion; linked with poor prognosis in cancer patients.	[126]
SNHG6	Up	miR-101-3p	acts as sponge for miR-101-3p, thereby upregulating ZEB1 at the post-transcriptional level and regulating E-cadherin; epigenetically inactivates P27 through EZH2-dependent histone H3 methylation in the promoter of the P27; activates the JNK pathway and upregulate P21	promotes EMT, invasion, migration, and metastasis	[65,127]
AF147447	Down	MUC2, miR-34c	acts as sponge for miR-34c, thus regulating MUC2, EGFR, and CD44 expression	suppresses EMT, cell invasion, and proliferation	[128]
SNHG5	Down	MTA2	provides a cytoplasmic trap for MTA2, directly binding to it and preventing its transfer from the cytoplasm into the nucleus	suppresses EMT, cell invasion, proliferation, and metastases	[129]
Linc00261	Down	SLUG	promotes SLUG degradation	suppresses EMT, cell invasion, and proliferation	[130]
AFAP1-AS1	Up	CDH1	upregulates E-cadherin and downregulates N-cadherin and vimentin	promotes EMT, invasion, and proliferation, associated with invasion in lymph nodes, distant metastasis, advanced TNM stages, and poor prognosis.	[131,132]
CASC15	Up	CDH1, miR-33a-5p, EZH2	targets CHD1; interacts with EZH2 and WDR5, recruits them to the CDKN1A promoter region, and thus modulates CDKN1A expression in the nucleus; acts as a sponge for miR-33a-5p and activates ZEB1 in the cytoplasm	promotes EMT, invasion, and proliferation, associated with poor prognosis	[133]
ZEB1-AS1	Up	miR-335-5p	downregulates miR-335-5p expression by acting as a molecular sponge	promotes EMT, invasion, and proliferation, correlates with lymph node metastasis, TNM stage, and poor overall survival of patients	[134,135]
NEAT1	Up	miR-506, CDH1	acts through the NEAT1/miR-506/STAT3 regulatory axis; targets CHD1	promotes EMT, invasion, and migration, correlates with more advanced stages, metastasis, and a low overall survival in patients	[136,137]
RP11-789C1.1	Down	miR-5003-3p	acts as sponge for miR-5003-3p	promotes migration, invasion, and EMT; correlates with metastases	[82,138]
LINC00675	Down	vimentin	regulates vimentin expression	suppresses proliferation, migration, invasion, and EMT	[139]
SOX2OT	Up	miR-194-5p	act as sponge for miR-194-5p	promotes EMT, cell proliferation, invasion, and migration	[140]
LINC01133	Down	miR-106a-3p	act as sponge for miR-106a-3p, which specifically targets the *APC*, and thus inactivates the Wnt/β-catenin pathway	inhibits proliferation, migration, EMT and metastasis	[141]
MEG3	Down	miR-21	act as sponge for miR-21; downregulating the expression of MMP-3, MMP-9, and VEGF; increases the expression of E-cadherin and downregulates the expression of N-cadherin, Snail, and β-catenin	suppresses EMT and cell mobility	[142]
SNHG14	Up	miR-145	negatively regulates miR-145 and thus affects its direct target; involved in PI3K/AKT/mTOR pathway	promotes EMT, cell viability, migration, invasion, and inhibits apoptosis	[143]

n/a—not available, EMT—Epithelial–Mesenchymal Transition.
